# Monotherapy versus combination therapy for multidrug-resistant Gram-negative infections: Systematic Review and Meta-Analysis

**DOI:** 10.1038/s41598-019-51711-x

**Published:** 2019-10-29

**Authors:** Adrian Schmid, Aline Wolfensberger, Johannes Nemeth, Peter W. Schreiber, Hugo Sax, Stefan P. Kuster

**Affiliations:** Division of Infectious Diseases and Hospital Epidemiology, University Hospital and University of Zurich, Zurich, Switzerland

**Keywords:** Bacterial infection, Risk factors

## Abstract

Infections caused by carbapenemase-producing, multidrug-resistant (MDR), or extensively drug-resistant (XDR) Gram-negative bacteria constitute a major therapeutic challenge. Whether combination antibiotic therapy is superior to monotherapy remains unknown. In this systematic review and meta-analysis OVID MEDLINE, EMBASE, PubMed, The Cochrane Library, and Scopus databases were searched for randomized controlled trials (RCTs) and observational studies published by December 2016 comparing mono- with combination antibiotic therapy for infections with carbapenemase-producing, MDR, or XDR Gram-negative bacteria. Mortality and clinical cure rates served as primary and secondary outcome measures, respectively. Of 8847 initially identified studies, 53 studies – covering pneumonia (n = 10 studies), blood stream (n = 15), osteoarticular (n = 1), and mixed infections (n = 27) - were included. 41% (n = 1848) of patients underwent monotherapy, and 59% (n = 2666) combination therapy. In case series/cohort studies (n = 45) mortality was lower with combination- vs. monotherapy (RR 0.83, CI 0.73–0.93, p = 0.002, I^2^ = 24%). Subgroup analysis revealed lower mortality with combination therapy with at least two *in-vitro* active antibiotics, in blood stream infections, and carbapenemase-producing Enterobacteriaceae. No mortality difference was seen in case-control studies (n = 6) and RCTs (n = 2). Cure rates did not differ regardless of study type. The two included RCTs had a high and unknown risk of bias, respectively. 16.7% (1/6) of case-control studies and 37.8% (17/45) of cases series/cohort studies were of good quality, whereas quality was poor in the remaining studies. In conclusion, combination antimicrobial therapy of multidrug-resistant Gram-negative bacteria appears to be superior to monotherapy with regard to mortality.

## Introduction

In an era of increasing antibiotic resistance rates, multidrug-resitant (MDR) Gram-negative bacteria constitute a major problem in the treatment of affected patients all over the world. The discovery void of new antibiotics, in particular antibiotics targeting Gram-negative bacteria, is further contributing to the problem and oftentimes necessitates the use of old drugs like polymyxins (colistin (polymyxin E) or polymyxin B), despite their well known and common serious side effects^1^. There is controversy on whether or not combination antimicrobial therapy is superior to monotherapy for infections with MDR pathogens. Many physicians prefer combination antimicrobial therapy over monotherapy, inferring superior outcomes mainly from *in vitro* experiments displaying synergistic effects of various antibiotics^2^. There are only few clinical studies comparing the effect of combination therapy versus monotherapy, most of them being retrospective cohort studies and case series limited by small study size. To account for the numerous confounding factors would require cohort studies including several hundreds to thousands of patients^3^. Furthermore, well-powered randomized controlled trials (RCTs) are lacking.

We conducted a systematic review and meta-analysis to investigate the effect of combination antimicrobial therapy versus monotherapy against multi-resistant Gram-negative bacteria on mortality as a primary outcome and cure rate as secondary outcome.

## Methods

### Data sources and searches

For this systematic review and meta-analysis, an electronic search of OVID MEDLINE, EMBASE, Scopus, The Cochrane Central Register of Controlled Trials (CENTRAL), and PubMed was performed with the help of a librarian with expertise in literature search for systematic reviews and meta-analyses. Studies published by December 2016 were included. For a detailed search strategy see Supplementary Materials (Supplementary Table 1).

### Study selection

RCTs, case-control studies, cohort studies and case series comparing outcomes of antibiotic monotherapy versus combination therapy of infections caused by carbapenemase-producing, MDR, XDR and PDR Gram-negative bacteria were included. We retained the definition of MDR and XDR used by respective study authors. Studies including not only carbapenemase-producing, MDR, XDR or PDR Gram-negative bacteria were excluded unless outcome data of infections caused by carbapenemase-producing, MDR, XDR or PDR bacteria were reported separately. Moreover, studies with inclusion of bacteria with resistance mechanisms to carbapenems other than carbapenemase production, studies reporting on patients under the age of 16 years, case series lacking a comparison group or including fewer than ten patients were excluded. No restriction by study site, country, follow-up period, dose, or frequency of drug application was applied. Only studies addressing parenteral and oral drug administration were included; antibiotics that got applied by inhalation or topically were not considered. Only studies published in English were considered eligible.

Titles and abstracts were screened and inclusion criteria applied by one author (AS). Full text articles of potentially relevant publications were obtained and reviewed independently by two out of three authors (AS, AW, SK) who took the decision on final inclusion by consensus. In case no agreement could be reached, the third author was consulted and final unanimous decision was taken after an in-depth discussion.

### Data extraction and quality assessment

Data extraction from included trials was performed by two of three authors (AS, AW, SK) using a standardized data collection form. In case of missing data, no attemps were made to contact study authors. Following data were extracted from studies: study design, microorganism and type of resistance, disease, antibiotics used in mono- and combination therapy and outcome measures. All-cause mortality and clinical cure rates, as defined in the individual studies, served as primary and secondary outcome measures, respectively. If the endpoints mortality or cure was only reported as probability, data were extracted as proportions.

In the monotherapy group, only patients treated with a single antibiotic drug were included, whereas in the combination therapy group patients treated with two or more antibiotics given simultaneously were included – regardless of *in vitro* susceptibility. Beta-lactamase inhibitors (i.e. sulbactam, clavulanic acid) were not considered independent antibiotic substances.

Quality assessment of RCTs with regard to method and risk of bias included: sequence generation, allocation concealment, blinding, incomplete outcome data, selective outcome reporting, and other sources of bias. Definitions/criteria were derived from the Cochrane Handbook for Systematic Reviews of Interventions^4^. Quality assessment and risk of bias of case-control studies comprised following criteria: selection, comparability, and exposure, according to the Newcastle-Ottawa quality assessment scale for case-control studies^5^. To assess quality and risk of bias of cohort studies, selection, comparability, and outcome was accounted for, using the Newcastle-Ottawa quality assessment scale for cohort studies^5^. Thresholds for converting the Newcastle-Ottawa scales to Agency for Healthcare Research and Quality (AHRQ) standards (good, fair and poor) were as follows: good quality if three or four stars in selection domain and one or two stars in comparability domain and two or three stars in outcome/exposure domain. Fair quality if two stars in selection domain and one or two stars in comparability domain and two or three stars in outcome/exposure domain. Poor quality if zero or one star in selection domain or zero stars in comparability domain or zero or one star in outcome/exposure domain^6^.

### Data synthesis and analysis

For Data synthesis and analysis, Review Manager (Version 5.3, The Cochrane Collaboration, The Nordic Cochrane Centre, Copenhagen, Denmark) was used. Due to anticipated heterogeneity between studies, random effects models were used for all analyses to obtain a summary estimate (risk ratio (RR)) of the average effect with its 95% confidence interval (CI). P-values ≤ 0.05 were considered statistically significant. Subgroup analyses for various resistance mechanisms, disease entities, antibiotics and quality of studies were performed. Statistical heterogeneity was initially inspected graphically using forest plots. The degree of heterogeneity was quantified by using the I^2^ statistic with a threshold of I^2^ > 60% for high heterogeneity. Publication bias was investigated using a funnel plot in which the standard error of the effect estimate of each study was plotted against the estimate.

## Results

### Study selection

**Figure 1:**
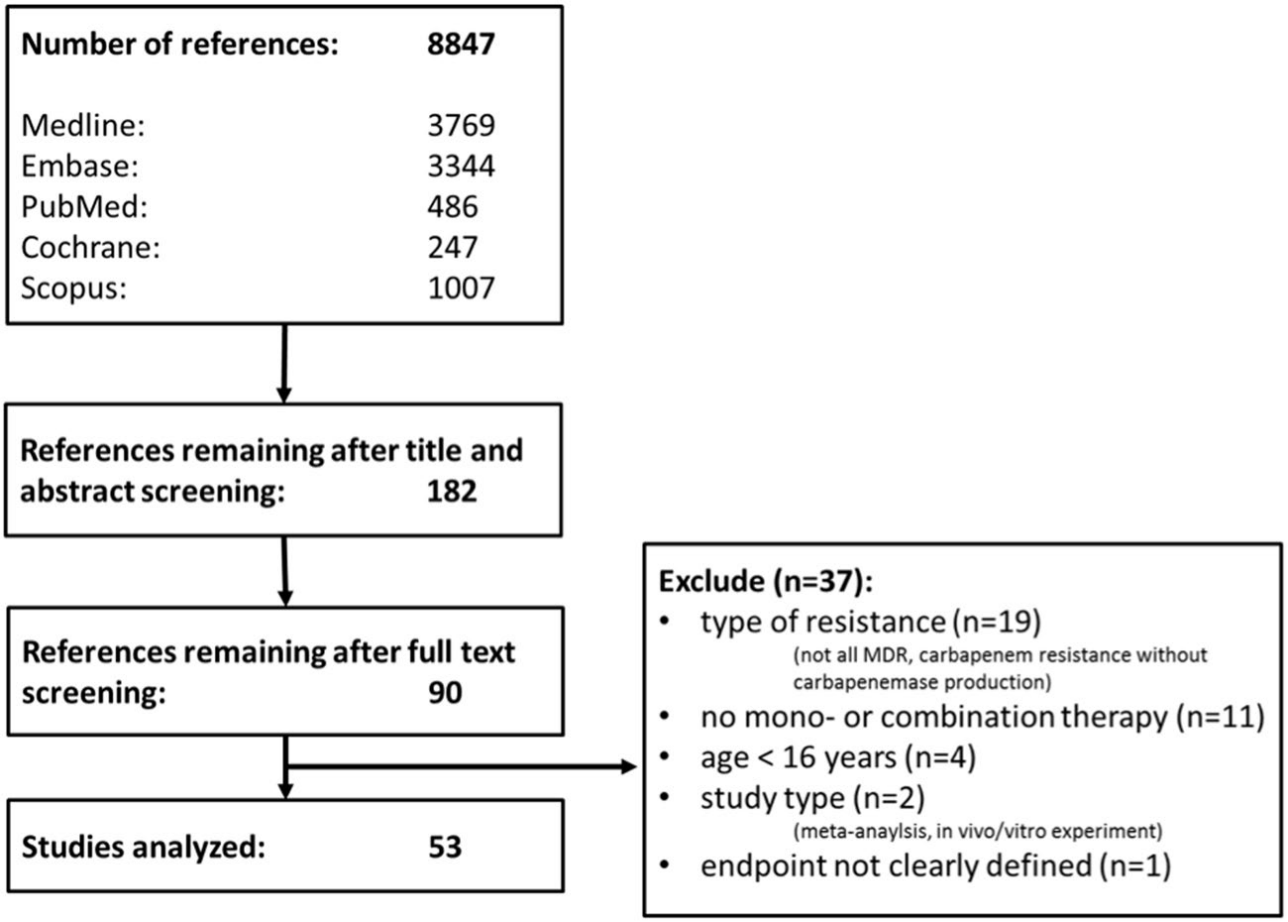
Study selection.

Figure 1 shows the study selection process according to the Preferred Reporting Items for Systematic reviews and Meta-Analyses for Protocols (PRISMA). The initial search yielded 8847 references. After screening titles and abstracts and exclusion of duplicates, 182 articles were selected for full-text screening, after which 53 studies were retained for the analysis. Included studies are depicted in Supplementary Table 2. Addressed infections ranged from pneumonia (n = 10 studies)^7–16^, blood stream infections (n = 15 studies)^17–31^, osteoarticular infections (n = 1 study)^32^, and studies comprising mixed forms of infections (n = 27 studies)^33–59^. Two of the included studies were RCTs^36,43^, six case-control studies^7,9,15,17,53,55^ and forty-five case series/cohort studies^8,10–14,16,18–35,37–42,44–52,54,56–59^. The studies included a total of 4514 patients: 41% of patients (n = 1848) were treated with monotherapy, 59% (n = 2666) with combination therapy.

Monotherapies comprised colistin, polymyxin B, minocycline, doxyxycline, carbapenem, tigecycline, beta-lactam/beta-lactamase inhibitor, cephalosporin, aminoglycoside, fosfomycin, nitrofurantoin, and fluoroquinolone. Combination therapy comprised colistin-based combinations, polymyxin B-based combinations, minocycline-based combinations, carbapenem-based combinations, tigecyclin-based combinations, and various other combinations. The following resistant bacteria were addressed by the studies: MDR *Pseudomonas aeruginosa*, Metallo-Beta-lactamase-producing *Pseudomonas aeruginosa*, MDR/XDR and pan-drug resistant (PDR) *Acinetobacter baumanii*, carbapenemase-producing Enterobacteriaceae (incl. KPC), and mixed MDR/XDR Gram-negatives.

### Mortality

All studies reporting on mortality totalled 2105 patients (56.3%) with combination therapy and 1633 patients (43.7%) with monotherapy.

### Case series and cohort studies

Meta-analysis of all case series and cohort studies included 1813 patients (56.7%) with combination therapy and 1383 patients (43.3%) with monotherapy and demonstrated a lower mortality risk in the combination therapy group (RR: 0.83, 95% CI 0.73–0.93, p = 0.002). Heterogeneity was low (I^2^ = 24%, p = 0.1).

**Figure 2:**
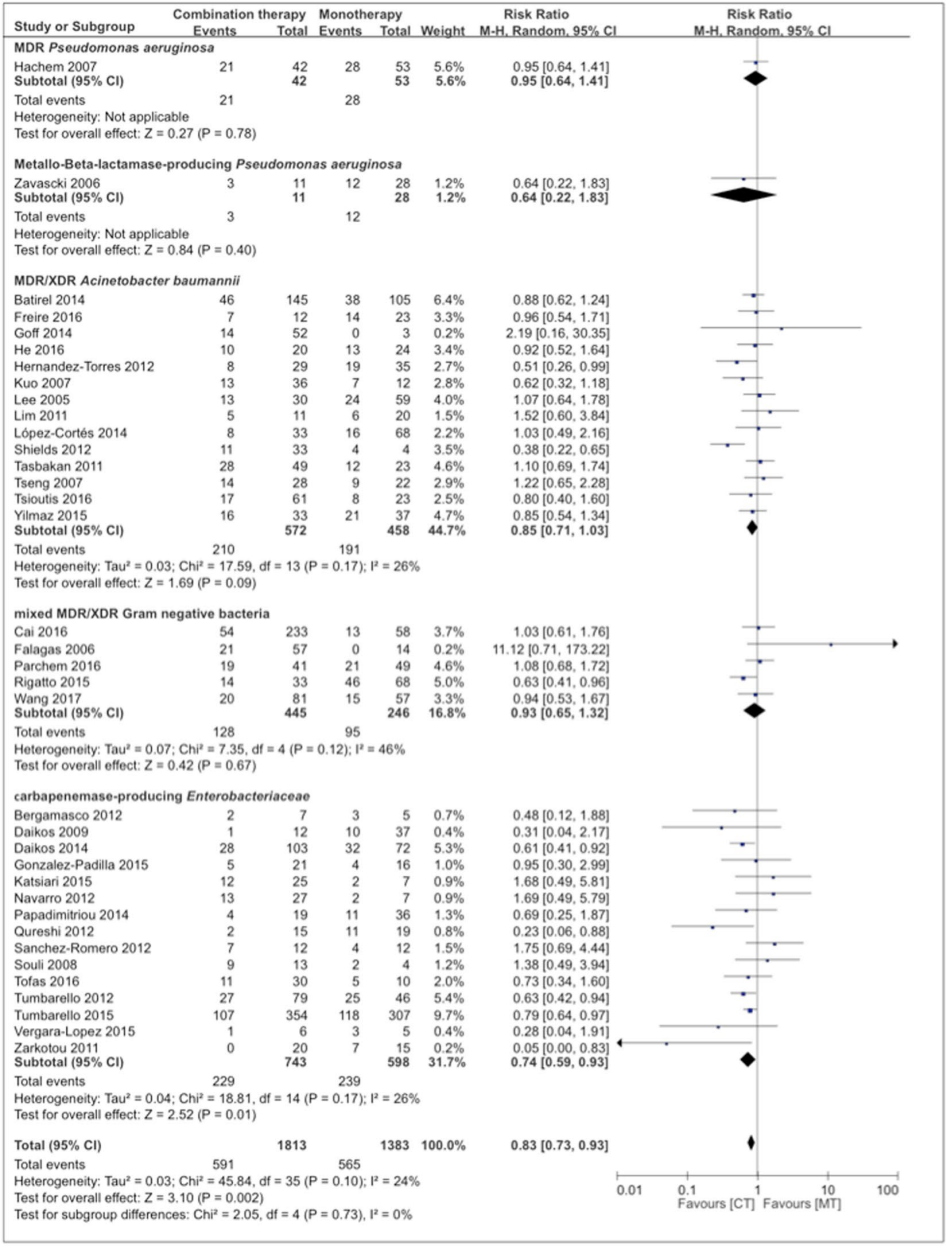
Risk ratios for mortality rates of case series and cohort studies stratified by different bacteria. Data markers indicate Risk ratios and error bars indicate 95% CIs.

Subgroup analysis of various bacteria and resistance mechanisms indicated a superior outcome of combination therapy versus monotherapy in the treatment of infections caused by carbapenemase-producing Enterobacteriaceae (RR 0.74, 95% CI 0.59–0.93, p = 0.01; I^2^ = 26%, p = 0.17). In infections caused by other resistant bacteria, i.e. MDR *Pseudomonas aeruginosa*, Metallo-Beta-lactamase-producing *Pseudomonas aeruginosa*, MDR/XDR *Acinetobacter baumanii* and mixed MDR/XDR Gram negative bacteria, no difference in mortality was detected (Fig. 2).

Subgroup analysis of various diseases revealed an advantage of combination therapy when applied in bloodstream infections (RR 0.78, 95% CI 0.63–0.96; p = 0.02; I^2^ = 27%, p = 0.17), whereas no difference could be seen in pneumonia or studies including mixed forms of infections. In studies that only included bloodstream infections (n = 14), stratified according to bacteria and resistance mechanism, combination therapy was superior in the group of carbapenemase-producing Enterobacteriaceae (RR: 0.61, CI 95% 0.45–0.85, p = 0.003; I^2^ = 21%, p = 0.26), with no difference in MDR/XDR *Acinetobacter baumanii* and mixed MDR/XDR Gram-negative bacteria (Supplementary Fig. 1). Subgroup analysis including only infections due to carbapenemase-producing Enterobacteriaceae, stratified by different disease types, again showed lower mortality rates in bloodstream infections (RR 0.61, CI 0.45–0.85, p = 0.003; I^2^ = 21%, p = 0.26) with no difference in the non-bloodstream infection group (comprising studies with mixed forms of infections) (Supplementary Fig. 2).

Subgroup analysis of different treatment strategies did not reveal a distinct combination therapy with a superior outcome compared to monotherapy. Analysis of the 12 studies with combination therapies that contained only one *in-vitro* active substance did not result in better outcomes against monotherapy, nor did subgroup analyses stratified according to bacteria and diseases. Polymyxin combinations performed better than polymyxin monotherapy (n = 1; RR 0.63, CI 95% 0.41–0.96, p = 0.03). Colistin combinations performed better than monotherapies with various antibiotics (n = 1; RR 0.38, CI 0.22–0.65, p = 0.0005).

**Figure 3:**
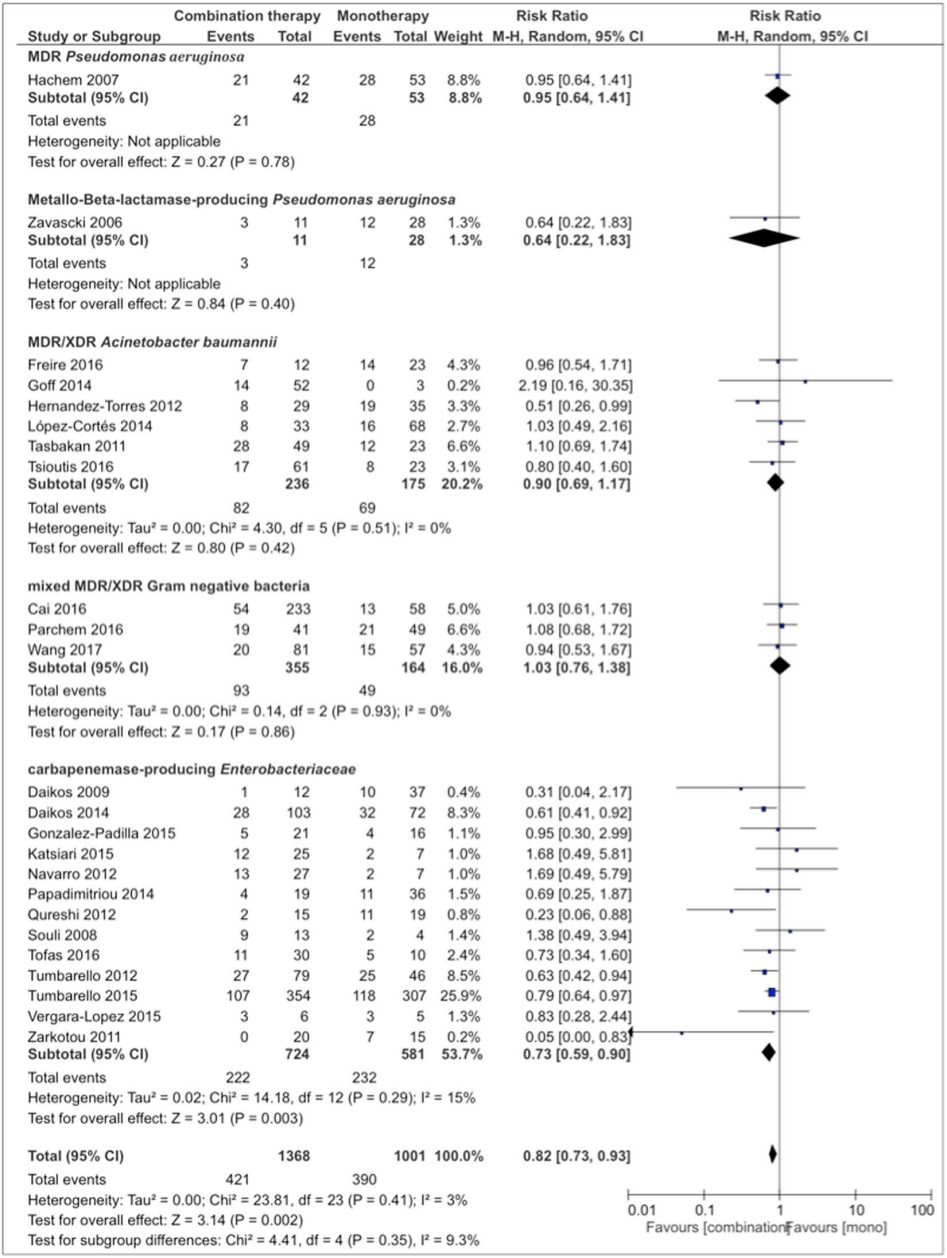
Risk ratios for mortality rates of case series and cohort studies stratified by different bacteria. Combination therapy with at least two *in vitro* active substances. Data markers indicate RRs and error bars indicate 95% CIs.

Meta-analysis of studies with combination therapy including at least two *in vitro* active substances (n = 24) showed lower mortality with combination therapy versus monotherapy (RR 0.82, CI 0.73–0.93, p = 0.002, I^2^ = 3%, p = 0.41) (Fig. 3). Subgroup analysis in this group revealed better results with carbapenemase-producing Enterobacteriaceae (RR 0.73, CI 0.59–0.90, p = 0.003; I^2^ = 15%, p = 0.29) and bloodstream infections (RR 0.70, CI 0.53–0.91, p = 0.009; I^2^ = 25%, p = 0.22).

Subgroup analysis of good and poor quality studies revealed a significant lower mortality risk with combination antibiotic therapy versus monotherapy in good quality studies (RR: 0.78, 95% CI 0.69–0.88, p = 0.0001, I^2^ = 4%, p = 0.4). Among studies with poor quality, no difference in mortality in the combination therapy vs. the monotherapy group was detected. With regard to the different diseases, a significant difference of combination antibiotic therapy was seen in good quality studies of blood stream infections (RR: 0.75, 95% CI 0.61–0.93, p = 0.009, I^2^ = 17%, p = 0.3) and good quality studies of mixed infections (RR: 0.77, 95% CI 0.64–0.92, p = 0.004, I^2^ = 5%, p = 0.38), whereas no advantage of combination therapy could be shown in poor quality studies. Subgroup analysis of different bacteria and quality of studies revealed a significant lower mortality with combination therapy of carbapenemase-producing Enterobacteriaceae in good quality studies (RR: 0.71, 95% CI 0.60–0.84, p < 0.0001, I^2^ = 0%, p = 0.49) in contrast to poor quality studies.

### Case-control studies

Meta-analysis of case-control studies comprising 142 patients (50.4%) with combination therapy and 140 patients (49.6%) with monotherapy did not show a significant difference in mortality between combination therapy and monotherapy. Neither did subgroup analyses of case-control studies comparing different bacteria/resistance mechanisms, diseases and treatments show a difference in mortality between the two groups. In all included case-control studies, either only one *in vitro* active substance was used in the combination group or it was not clearly stated whether there were several *in-vitro* active substances in the combination group.

The only study of good quality revealed a lower mortality of combination therapy (RR: 0.22, 95% CI 0.09–0.56, p = 0.001). Poor quality studies did not show any difference between combination therapy and monotherapy.

### Randomized controlled trials

Meta-analysis of the two RCTs (150 patients with combination treatment, 110 patients with monotherapy) and various subgroup analyses did not show a beneficial effect of combination therapy over monotherapy. In the combination groups of the included RCTs, two or more *in-vitro* active substances were used.

### Clinical cure rates

For meta-analysis of clinical cure rates, data from 1666 patients were available including case series, cohort and case-control studies. Among these, 1069 patients (64.2%) received combination therapy and 597 patients (35.8%) were treated with a monotherapy.

### Case series and cohort studies

**Figure 4:**
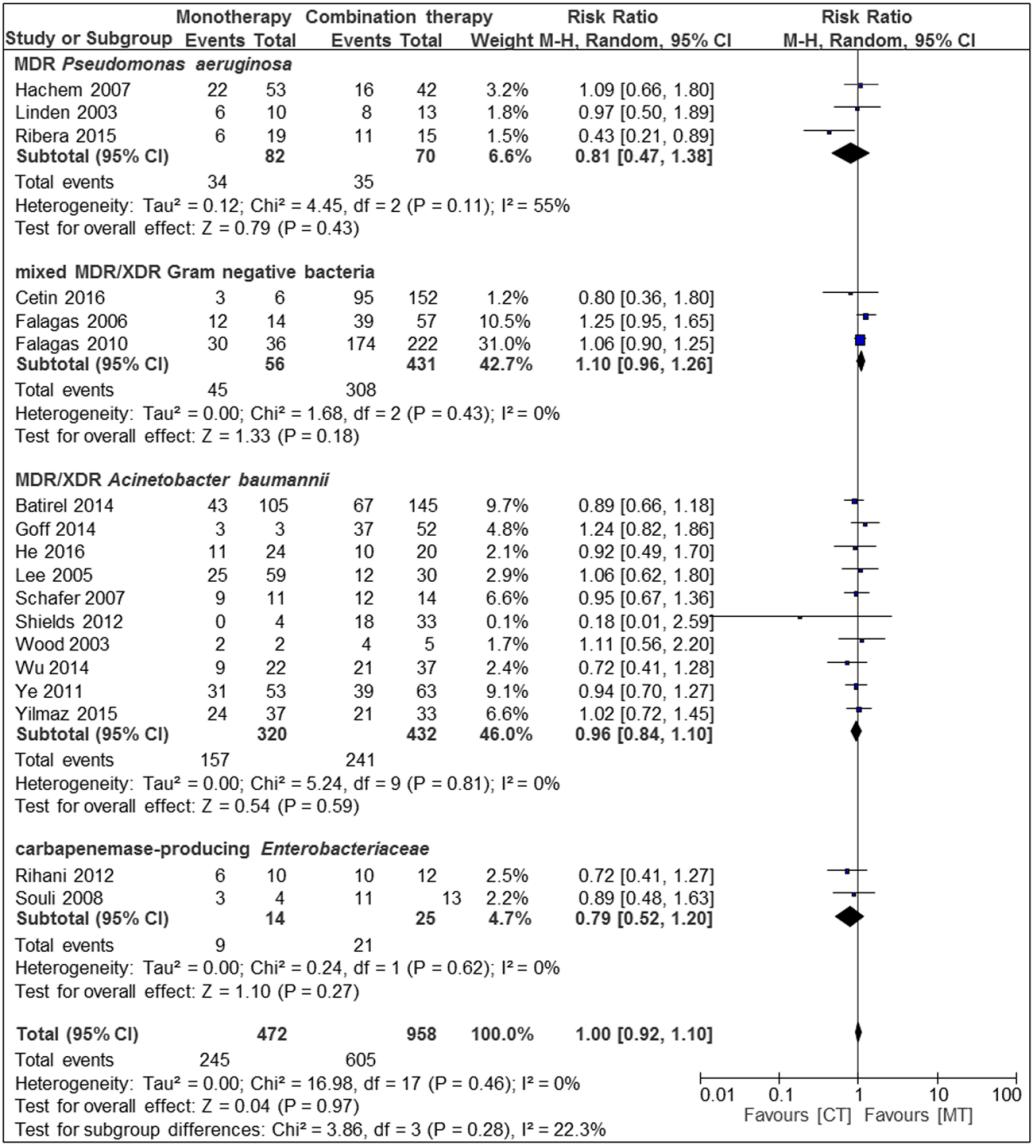
Risk ratios for clinical cure rates of case series and cohort studies stratified by different bacteria. Data markers indicate Risk ratios and error bars indicate 95% CIs.

Meta-analysis of case series and cohort studies with a total of 958 patients (67.0%) with combination therapy and 472 patients (33.0%) with monotherapy, did not show a difference in clinical cure rates between mono- and combination therapy (Fig. 4).

Subgroup analysis of different bacteria, resistance mechanisms and quality of studies did not reveal a difference between mono- and combination therapy. A trend to higher cure rates in combination therapy was seen in MDR *Pseudomonas aeruginosa*, MDR *Acinetobacter baumanii* and carbapenemase-producing Enterobacteriaceae.

Subgroup analysis of different diseases revealed a difference between mono- and combination therapy in osteoarticular infection (n = 1; RR 0.43, CI 0.21–0.89, p = 0.02) but no difference in other types of infections (pneumonia, bloodstream infections, mixed forms of infection).

**Figure 5:**
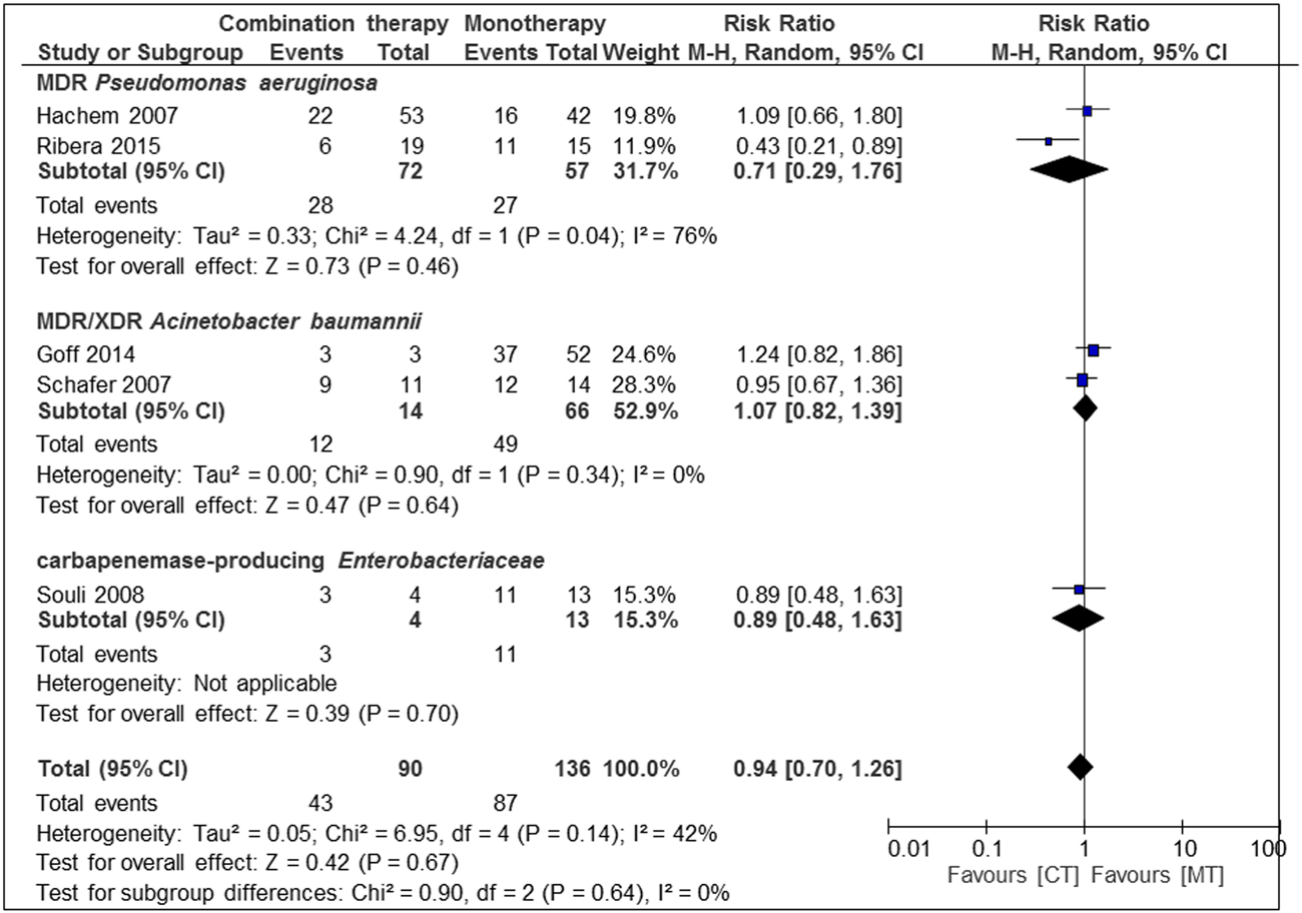
Risk ratios for clinical cure rates of case series and cohort studies stratified by different bacteria. Combination therapy with at least two *in vitro* active substances. Data markers indicate Risk ratios and error bars indicate 95% CIs.

No difference in cure rates was seen with different antibiotic therapies, regardless of the number of substances with *in-vitro* activity in combination treatment groups (Fig. 5).

### Case-control studies

Meta-analysis of case-control studies, including 125 patients (53.0%) with mono- and 111 patients (47.0%) with combination therapy, did not show any difference of monotherapy vs. combination therapy with regard to clinical cure rates. Subgroup analyses looking at different bacteria/resistance mechanisms, diseases (pneumonia, mixed) and treatments did not show any difference in clinical cure rates.

### Study quality

The quality of included studies was variable. Two of the included studies were RCTs, one of which was un-blinded and the other one did not report on blinding leading to potential performance bias. One RCT showed an overall high risk of bias and the second one an overall unknown risk of bias (Supplementary Table 3). Only one out of six included case-control studies (16.7%) and 17 out of 45 (37.8%) included cases series and cohort studies showed an overall good quality with good comparability of cases and controls. The remaining studies were prone to confounding factors resulting in poor quality (Supplementary Table 4 and Supplementary Table 5). Funnel plots did not suggest publication bias (data not shown).

## Discussion

In this large meta-analysis including 53 studies with a total of 4514 patients, overall mortality in case series and cohort studies was lower in patients treated with antibiotic combination therapy. Specifically, lower mortality rates were detected in patients with bloodstream infections and in infections caused by carbapenemase-producing Enterobacteriacae if combination therapy entailed at least two substances with *in-vitro* activity. There was no difference in mortality rates if combination therapy comprised only one *in-vitro* active substance, in RCTs (2 studies; several *in-vitro* active substances in combination therapy) and case-control studies (5 studies; only one *in-vitro* active substance in combination therapy). These effects were confirmed when restricting our analyses to studies of good quality.

Eight^19,20,24,25,27,29,31^ out of fourteen case series and cohort studies (57%) that included patients with bloodstream infections were due to carbapenemase-producing Enterobacteriaceae. These studies also accounted for 53% (8/15) of all included case series and cohort studies due to carbapenemase-producing Enterobacteriaceae. The mortality rate of patients with bloodstream infections with bacteria other than carbapenemase-producing Enterobacteriaceae did not differ between combination and monotherapy. Likewise, non-bloodstream infections by carbapenemase-producing Enterobacteriaceae did not show a superior effect of combination therapy. Thus, it is likely that the superior effect of combination therapy with infections caused by carbapenemase-producing Enterobacteriaceae and with bloodstream infections is actually one and the same.

Our results are in line with published results by Zusman *et al*.^60^, who showed lower mortality with polymyxin/carbapenem or polymyxin/tigecycline, aminoglycosides or fosfomycin combination therapy compared to polymyxin monotherapy in carbapenem-resistant bacteria and could also show a benefit of combined treatment in bloodstream infections. In contrast to our meta-analysis, Zusman *et al*. included carbapenem-resistant bacteria in general (i.e. also including carbapenem resistance due to porin loss), and regarded betalactamase inhibitors (i.e. sulbactam) as separate antibiotic substances. Furthermore, Zusman *et al*. only included carbapenem-resistant bacteria and only polymyxin mono- and combination therapy, whereas our analysis comprised different multiresistant bacteria and antimicrobials.

In theory, several facts argue against the application of combination therapy: the possible rise in resistance rates due to an overall increase in selection pressure as a result of a greater release of antibiotics into the environment^61^, higher rates of adverse effects (such as nephro- and ototoxicity due to colistin or aminoglycosides)^61^, an increase in *Clostridium difficile*-associated infections, fungal infections^3^, higher costs^61^, and possible antagonism^3^. On the other hand, several factors argue in favour of combination therapy: a broader antibiotic spectrum, *in vitro* data showing synergy of antibiotic combinations^2^, lower efficacy of colistin monotherapy compared to betalactam monotherapy^62^, lower risk of resistance development (e.g. against colistin)^1^, lower doses and shorter treatment duration^61^.

Observational study designs are prone to several limitations, mainly due to unmeasured confounding factors and other risks of bias. The likelihood of inappropriate empirical antibiotic therapy is higher in the monotherapy group and the time to start of appropriate monotherapy after results from susceptibilty testing are known may be longer compared to the combination therapy group, which may result in a bias in favour of combination therapy^3^. Information about the dosing of colistin was only reported in a minority of the included studies^12,27,29,34^. As guidance has changed in recent years and a loading dose is now generally recommended, colistin could have been underdosed in a significant proportion of studies, which may constitute a bias against colistin monotherapy^3^. Furthermore, there is a chance of selection bias in favour of combination therapy, as patients are more likely to receive colistin or tigecyclin monotherapy if bacteria are resistant to all other antibiotics as compared to situations where bacteria are still susceptible to carbapenems, for example, or in patients who suffer from polymicrobial infections with resistant as well as susceptible strains^3^. Such an effect could result in a perceived lower overall mortality in the combination treatment group at least partly due to a subgroup of carbapenem susceptible Gram-negative bacteria^20,29,56^. Moreover, combination therapy is more likely prescribed to patients with polymicrobial infections in which one does not know for sure whether the resistant bacterium is the main causative agent^3^. Against the latter argues the fact that an advantage of combination therapy could be shown in bloodstream infections, where one can almost be certain of the main causative agent, in analogy to Zusman and colleagues^60^. Finally, one could argue that combination therapy is more likely to be prescribed in more serious infections, which would bias the results in favour of monotherapy.

Due to numerous limitations and biases, which is reflected in the quality assessment of included studies, the results of this metaanalysis have to be interpreted with caution. However, by inclusion of a wide variety of cohort studies and case series, including a heterogeneous group of infectious diseases, bacteria, and treatments, we tried to minimize existing biases and limitations and allow for a valid comparison of combination treatment with monotherapy. Moreover, the favourable effect of combination therapy in bloodstream infections due to carbapenmase-producing Enterobacteriaceae has recently been confirmed in a large retrospective cohort for the subset of patients with severe infections^63^. With regard to RCTs and case-control studies, the validity of the results of our meta-analysis of RCTs and case-control studies is very limited due to the low number and limited power of the included studies.

To reach a final conclusion as to the possible advantage of combination therapy versus monotherapy, well designed and sufficiently powered RCTs are needed. Two RCT’s currently being undertaken could bring some more clarity in this contentious issue^64,65^. Such trials, however, will always have the problem to be prone to the conclusion that their findings can only be applied to the population under study. Only pragmatic trial designs will help resolve this issue. For the time being and taking into account limiting factors of RCTs, such as problems in reaching sufficient power in the setting of uncommon diseases as well as costs, other forms of study (cohort studies, case series, case-control studies) provide valid and valuable data on the best evidence-based treatment^66^. Individual patient-data meta-analyses may add further insights when it proves to be difficult to obtain large enough sample sizes for certain study questions.

Based on our results, combination therapy with two antimicrobials that demonstrate *in vitro* activity should be administered in patients with bloodstream infections due to carbapenemase-producing Enterobacteriaceae.

## Supplementary information

Supplementary Information
