# Supplementary material for: Monotherapy versus combination therapy for multidrug-resistant Gram-negative infections: Systematic Review and Meta-Analysis
Source: Sci Rep. 2019 Oct 29;9:15290. doi: 10.1038/s41598-019-51711-x (PMC6821042; doi:10.1038/s41598-019-51711-x)
Supplement: Supplementary file 1 — Supplementary Information [file 41598_2019_51711_MOESM1_ESM.docx]

Adrian Schmid MD^1^, Aline Wolfensberger MD^1^, Johannes Nemeth MD^1^, Peter W. Schreiber MD^1^, Prof Hugo Sax MD^1^, Stefan P. Kuster MD^1^

**Monotherapy versus combination therapy for multidrug-resistant Gram-negative infections: Systematic Review and Meta-Analysis**

**SUPPLEMENTARY INFORMATION**

| **Scopus** | 1 | ( ( ( TITLE-ABS-KEY ( ( acetobacteraceae OR achromobacter OR alcaligenes OR alteromonadaceae OR azorhizobium OR bdellovibrio OR beggiatoa OR bordetella OR brucella OR burkholderiaceae OR caulobacteraceae OR chryseobacterium OR elizabethkingia OR flavobacterium OR francisella OR halomonas OR legionellaceae OR leptothrix OR methylobacillus OR methylobacterium OR methylococcaceae OR methylophilus OR methylosinus OR neisseriaceae OR ochrobactrum OR paracoccus OR pseudomonadaceae OR rhizobiaceae OR taylorella OR thermus OR xanthobacter OR acidaminococcaceae OR bacteroides OR bilophila OR butyrivibrio OR desulfovibrio OR dichelobacter OR fusobacterium OR oxalobacter OR porphyromonas OR prevotella OR thauera OR thermotoga OR wolinella OR hyphomicrobium OR nitrobacter OR nitrosomonadaceae OR nitrosospira OR aeromonadaceae OR chromobacterium OR eikenella OR enterobacteriaceae OR pasteurellaceae OR shewanella OR sphingomonas OR streptobacillus OR vibrionaceae OR zymomonas OR lawsonia OR acidithiobacillus OR afipia OR alcaligenaceae OR alteromonas OR azorhizobium OR bdellovibrio OR bradyrhizobiaceae OR bradyrhizobium OR brucellaceae OR burkholderiaceae OR caulobacteraceae OR comamonadaceae OR coxiellaceae OR cytophagaceae OR flavobacteriaceae OR francisella OR gluconacetobacter OR halomonadaceae OR halothiobacillus OR klebsiella OR legionellaceae OR leptospiraceae OR methylobacteriaceae OR methylococcaceae OR methylophilaceae OR moraxellaceae OR acinetobacter OR moraxella OR psychrobacter OR neisseriaceae OR nitrosomonadaceae OR ochrobactrum OR oxalobacteraceae OR paracoccus OR pedobacter OR pseudoalteromonas OR pseudomonadaceae OR rhizobiaceae OR rhodospirillaceae OR rhodothermus OR sphingobacterium OR sphingomonas OR thermus OR xanthobacter OR xanthomonadaceae OR zoogloea OR thiobacillus OR gramnegative OR "gram negative" OR gram-negative ) W/6 infect* ) ) AND ( ( TITLE-ABS-KEY ( ( ( antibiotic OR multidrug OR drug OR carbapenem ) W/3 resistan* ) ) ) OR ( TITLE-ABS-KEY ( ( bacteria* OR bacill* OR microbial OR baumannii ) W/3 resistan* ) ) OR ( TITLE-ABS-KEY ( ( acetobacteraceae OR achromobacter OR alcaligenes OR alteromonadaceae OR azorhizobium OR bdellovibrio OR beggiatoa OR bordetella OR brucella OR burkholderiaceae OR caulobacteraceae OR chryseobacterium OR elizabethkingia OR flavobacterium OR francisella OR halomonas OR legionellaceae OR leptothrix OR methylobacillus OR methylobacterium OR methylococcaceae OR methylophilus OR methylosinus OR neisseriaceae OR ochrobactrum OR paracoccus OR pseudomonadaceae OR rhizobiaceae OR taylorella OR thermus OR xanthobacter OR acidaminococcaceae OR bacteroides OR bilophila OR butyrivibrio OR desulfovibrio OR dichelobacter OR fusobacterium OR oxalobacter OR porphyromonas OR prevotella OR thauera OR thermotoga OR wolinella OR hyphomicrobium OR nitrobacter OR nitrosomonadaceae OR nitrosospira OR aeromonadaceae OR chromobacterium OR eikenella OR enterobacteriaceae OR pasteurellaceae OR shewanella OR sphingomonas OR streptobacillus OR vibrionaceae OR zymomonas OR lawsonia OR acidithiobacillus OR afipia OR alcaligenaceae OR alteromonas OR azorhizobium OR bdellovibrio OR bradyrhizobiaceae OR bradyrhizobium OR brucellaceae OR burkholderiaceae OR caulobacteraceae OR comamonadaceae OR coxiellaceae OR cytophagaceae OR flavobacteriaceae OR francisella OR gluconacetobacter OR halomonadaceae OR halothiobacillus OR klebsiella OR legionellaceae OR leptospiraceae OR methylobacteriaceae OR methylococcaceae OR methylophilaceae OR moraxellaceae OR acinetobacter OR moraxella OR psychrobacter OR neisseriaceae OR nitrosomonadaceae OR ochrobactrum OR oxalobacteraceae OR paracoccus OR pedobacter OR pseudoalteromonas OR pseudomonadaceae OR rhizobiaceae OR rhodospirillaceae OR rhodothermus OR sphingobacterium OR sphingomonas OR thermus OR xanthobacter OR xanthomonadaceae OR zoogloea OR thiobacillus OR gramnegative OR "gram negative" OR gram-negative ) W/3 resistan* ) ) OR ( TITLE-ABS-KEY ( carbapenemase* W/3 produc* ) ) ) ) AND ( TITLE-ABS- KEY ( ( drug OR medic* ) W/3 ( therapy OR treatment ) ) OR TITLE ( treatment OR therapy ) ) ) AND ( TITLE-ABS-KEY ( ( cure* OR surviv* OR heal* OR outcome OR mortality OR death* OR safety OR effectiv* OR success* ) ) ) AND ( LIMIT-TO ( LANGUAGE , "English" ) OR LIMIT-TO ( LANGUAGE , "German" ) ) |
| --- | --- | --- |
| **OVID Medline** | 1 | exp Gram-Negative Bacterial Infections |
|  | 2 | ((Acetobacter* or Achromobacter* or Alcaligene* or Alteromonadac* or Azorhizobium or Bdellovibrio or Beggiatoa or Bordetella orBrucella or Burkholderiacea* or Caulobacteracea* or Chryseobacterium or Elizabethkingia or Flavobacterium or Francisella orHalomonas or Legionellacea* or Klebsiella or Leptothrix or Methylobacill* or Methylobacter* or Methylococcacea* or Methylophil* orMethylosin* or Neisseriacea* or Ochrobactrum or Paracocc* or Pseudomonadacea* or Rhizobiacea* or Taylorell* or Thermus orXanthobacter or Acidaminococcacea* or Bacteroides or Bilophila or Butyrivibrio or Desulfovibrio or Dichelobacter or Fusobacterium orOxalobacter or Porphyromona* or Prevotella or Thauera or Thermotoga or Wolinella or Hyphomicrobi* or Nitrobacter orNitrosomonadacea* or Nitrosospira or Aeromonadacea* or Chromobacterium or Eikenella or Enterobacteriacea* or Pasteurellacea* orShewanella or Sphingomonas or Streptobacill* or Vibrionacea* or Zymomonas or Lawsonia or Acidithiobacillus or Afipia orAlcaligenaceae or Alteromonas or Azorhizobium or Bdellovibrio or Bradyrhizobiaceae or Bradyrhizobium or Brucellaceae orBurkholderiaceae or Caulobacteraceae or Comamonadaceae or Coxiellaceae or Cytophagaceae or Flavobacteriaceae or Francisella orGluconacetobacter or Halomonadaceae or Halothiobacillus or Legionellaceae or Leptospiraceae or Methylobacteriaceae orMethylococcaceae or Methylophilaceae or Moraxellaceae or Acinetobacter or Moraxella or Psychrobacter or Neisseriaceae orNitrosomonadaceae or Ochrobactrum or Oxalobacteraceae or Paracoccus or Pedobacter or Pseudoalteromonas orPseudomonadaceae or Rhizobiaceae or Rhodospirillaceae or Rhodothermus or Sphingobacterium or Sphingomonas orThermus orXanthobacter or Xanthomonadaceae or Zoogloea or Thiobacillusor gramnegative or "gram negative" or gram-negative) adj6infect*).ti,ab. |
|  | 3 | exp *Gram-Negative Bacteria/ |
|  | 4 | exp Bacterial Infections/ |
|  | 5 | 3 and 4 |
|  | 6 | 1 or 2 or 5 |
|  | 7 | exp drug resistance, bacterial/ or drug resistance, multiple, bacterial/ |
|  | 8 | ((antibiotic or multidrug or drug or carbapenem) adj3 resistan*).ti,ab. |
|  | 9 | ((bacteria* or bacill* or microbial or baumannii) adj3 resistan*).ti,ab. |
|  | 10 | ((Acetobacter* or Achromobacter* or Alcaligene* or Alteromonadac* or Azorhizobium or Bdellovibrio or Beggiatoa or Bordetella orBrucella or Burkholderiacea* or Caulobacteracea* or Chryseobacterium or Elizabethkingia or Flavobacterium or Francisella orHalomonas or klebsiella or Legionellacea* or Leptothrix or Methylobacill* or Methylobacter* or Methylococcacea* or Methylophil* orMethylosin* or Neisseriacea* or Ochrobactrum or Paracocc* or Pseudomonadacea* or Rhizobiacea* or Taylorell* or Thermus orXanthobacter or Acidaminococcacea* or Bacteroides or Bilophila or Butyrivibrio or Desulfovibrio or Dichelobacter or Fusobacterium orOxalobacter or Porphyromona* or Prevotella or Thauera or Thermotoga or Wolinella or Hyphomicrobi* or Nitrobacter orNitrosomonadacea* or Nitrosospira or Aeromonadacea* or Chromobacterium or Eikenella or Enterobacteriacea* or Pasteurellacea* orShewanella or Sphingomonas or Streptobacill* or Vibrionacea* or Zymomonas or Lawsonia or Acidithiobacillus or Afipia orAlcaligenaceae or Alteromonas or Azorhizobium or Bdellovibrio or Bradyrhizobiaceae or Bradyrhizobium or Brucellaceae orBurkholderiaceae or Caulobacteraceae or Comamonadaceae or Coxiellaceae or Cytophagaceae or Flavobacteriaceae or Francisella orGluconacetobacter or Halomonadaceae or Halothiobacillus or Legionellaceae or Leptospiraceae or Methylobacteriaceae orMethylococcaceae or Methylophilaceae or Moraxellaceae or Acinetobacter or Moraxella or Psychrobacter or Neisseriaceae orNitrosomonadaceae or Ochrobactrum or Oxalobacteraceae or Paracoccus or Pedobacter or Pseudoalteromonas orPseudomonadaceae or Rhizobiaceae or Rhodospirillaceae or Rhodothermus or Sphingobacterium or Sphingomonas orThermus orXanthobacter or Xanthomonadaceae or Zoogloea or Thiobacillusor gramnegative or "gram negative" or gram-negative) adj3resistan*).ti,ab. |
|  | 11 | (carbapenemase* adj3 produc*).ti,ab. |
|  | 12 | or/7-11 |
|  | 13 | 6 and 12 |
|  | 14 | exp Gram-Negative Bacterial Infections/dt, th [Drug Therapy, Therapy] |
|  | 15 | exp Drug Therapy/ |
|  | 16 | Drug Therapy.fs. |
|  | 17 | ((drug or medic*) adj3 (therapy or treatment)).ab. |
|  | 18 | (therapy or treatment).ti. |
|  | 19 | or/14-18 |
|  | 20 | 13 and 19 |
|  | 21 | exp Hospital Mortality/ or exp Mortality/ or mortality.mp. |
|  | 22 | exp Survival Analysis/ or exp Survival/ or survival.mp. or exp Survival Rate/ |
|  | 23 | exp Treatment Outcome/ |
|  | 24 | (cure* or surviv* or heal* or outcome or mortality or death* or safety or effectiv* or success*).ti,ab. |
|  | 25 | or/21-24 |
|  | 26 | 20 and 25 |
|  | 27 | 26 not (animals not humans).sh. |
|  | 28 | limit 27 to (english or german) |
| **Embase** | #1 | 'gram negative infection'/exp |
|  | #2 | gram negative bacterium'/exp/mj |
|  | #3 | bacterial infection'/exp |
|  | #4 | #2 AND #3 |
|  | #5 | ((acetobacter* OR achromobacter* OR alcaligene* OR alteromonadac* OR azorhizobium OR bdellovibrio OR beggiatoa OR bordetella OR brucella OR burkholderiacea* OR caulobacteracea* OR chryseobacterium OR elizabethkingia OR flavobacterium OR francisella OR halomonas OR legionellacea* OR klebsiella OR leptothrix OR methylobacill* OR methylobacter* OR methylococcacea* OR methylophil* methylosin* OR neisseriacea* OR ochrobactrum OR paracocc* OR pseudomonadacea* OR rhizobiacea* OR taylorell* OR thermus OR xanthobacter OR acidaminococcacea* OR bacteroides OR bilophila OR butyrivibrio OR desulfovibrio OR dichelobacter fusobacterium OR oxalobacter OR porphyromona* OR prevotella OR thauera OR thermotoga OR wolinella OR hyphomicrobi* OR nitrobacter OR nitrosomonadacea* OR nitrosospira OR aeromonadacea* OR chromobacterium OR eikenella OR enterobacteriacea* OR pasteurellacea* OR shewanella OR sphingomonas OR streptobacill* OR vibrionacea* OR zymomonas OR lawsonia OR acidithiobacillus OR afipia OR alcaligenaceae OR alteromonas OR azorhizobium OR bdellovibrio OR bradyrhizobiaceae OR bradyrhizobium OR brucellaceae OR burkholderiaceae OR caulobacteraceae OR comamonadaceae OR coxiellaceae OR cytophagaceae OR flavobacteriaceae OR francisella OR gluconacetobacter OR halomonadaceae OR halothiobacillus OR legionellaceae OR leptospiraceae OR methylobacteriaceae OR methylococcaceae OR methylophilaceae OR moraxellaceae OR acinetobacter OR moraxella OR psychrobacter OR neisseriaceae OR nitrosomonadaceae OR ochrobactrum OR oxalobacteraceae OR paracoccus OR pedobacter OR pseudoalteromonas OR pseudomonadaceae OR rhizobiaceae OR rhodospirillaceae OR rhodothermus OR sphingobacterium OR sphingomonas OR thermus OR xanthobacter OR xanthomonadaceae OR zoogloea OR thiobacillus OR gramnegative OR 'gram negative' OR 'gram negative' ) NEAR/6 infect*):ab,ti |
|  | #6 | #1 OR #4 OR #5 |
|  | #7 | 'antibiotic resistance'/exp |
|  | #8 | ((antibiotic OR multidrug OR drug OR carbapenem) NEAR/3 resistan*):ab,ti |
|  | #9 | ((bacteria* OR bacill* OR microbial OR baumannii) NEAR/3 resistan*):ab,ti |
|  | #10 | ((acetobacter* OR achromobacter* OR alcaligene* OR alteromonadac* OR azorhizobium OR bdellovibrio OR beggiatoa OR bordetella OR brucella OR burkholderiacea* OR caulobacteracea* OR chryseobacterium OR elizabethkingia OR flavobacterium OR francisella OR halomonas OR klebsiella OR legionellacea* OR leptothrix OR methylobacill* OR methylobacter* OR methylococcacea* OR methylophil* OR methylosin* OR neisseriacea* OR ochrobactrum OR paracocc* OR pseudomonadacea* OR rhizobiacea* OR taylorell* OR thermus OR xanthobacter OR acidaminococcacea* OR bacteroides OR bilophila OR butyrivibrio OR desulfovibrio OR dichelobacter OR fusobacterium OR oxalobacter OR porphyromona* OR prevotella OR thauera OR thermotoga OR wolinella OR hyphomicrobi* OR nitrobacter OR nitrosomonadacea* OR nitrosospira OR aeromonadacea* OR chromobacterium OR eikenella OR enterobacteriacea* OR pasteurellacea* OR shewanella OR sphingomonas streptobacill* OR vibrionacea* OR zymomonas OR lawsonia OR acidithiobacillus OR afipia OR alcaligenaceae OR alteromonas OR azorhizobium OR bdellovibrio OR bradyrhizobiaceae OR bradyrhizobium OR brucellaceae OR burkholderiaceae OR caulobacteraceae OR comamonadaceae OR coxiellaceae OR cytophagaceae OR flavobacteriaceae OR francisella OR gluconacetobacter OR halomonadaceae OR halothiobacillus OR legionellaceae OR leptospiraceae OR methylobacteriaceae OR methylococcaceae OR methylophilaceae OR moraxellaceae OR acinetobacter OR moraxella psychrobacter OR neisseriaceae OR nitrosomonadaceae OR ochrobactrum OR oxalobacteraceae OR paracoccus OR pedobacter OR pseudoalteromonas OR pseudomonadaceae OR rhizobiaceae OR rhodospirillaceae OR rhodothermus OR sphingobacterium OR sphingomonas OR thermus OR xanthobacter OR xanthomonadaceae OR zoogloea OR thiobacillus OR gramnegative OR 'gram negative' OR 'gram negative' ) NEAR/3 resistan *):ab,ti |
|  | #11 | (carbapenemase* NEAR/3 produc*):ab,ti |
|  | #12 | #7 OR #8 OR #9 OR #10 OR #11 |
|  | #13 | #6 AND #12 |
|  | #14 | 'gram positive infection'/exp/dm_dr |
|  | #15 | #13 OR #14 |
|  | #16 | 'gram positive infection'/exp/dm_dt |
|  | #17 | 'drug therapy'/exp |
|  | #18 | ((drug OR medic*) NEAR/3 (therapy OR treatment)):ab,ti |
|  | #19 | treatment:ti OR threapy:ti |
|  | #20 | 'drug therapy':lnk |
|  | #21 | #16 OR #17 OR #18 OR #19 OR #20 |
|  | #22 | #15 AND #21 |
|  | #23 | 'mortality'/exp OR 'survival'/exp OR 'treatment outcome'/exp OR 'convalescence'/exp |
|  | #24 | cure*:ab,ti OR surviv*:ab,ti OR heal*:ab,ti OR outcome:ab,ti OR mortality:ab,ti OR death*:ab,ti OR safety:ab,ti OR effectiv*:ab,ti OR success*:ab,ti |
|  | #25 | #23 OR #24 |
|  | #26 | #22 AND #25 NOT ([animals]/lim NOT [humans]/lim) AND ([english]/lim OR [german]/lim) |
|  | #27 | #22 AND #25 NOT ([animals]/lim NOT [humans]/lim) AND ([english]/lim OR [german]/lim) |
|  | #28 | #22AND #25 NOT ([animals]/lim NOT [humans]/lim) AND ([english]/lim OR [german]/lim) AND [conference abstract]/lim |
|  | #29 | #22AND #25 NOT ([animals]/lim NOT [humans]/lim) AND ([english]/lim OR [german]/lim) NOT [conference abstract]/lim |
| **PubMed** |  | Search (((((((((((((Acetobacteraceae OR Achromobacter OR Alcaligenes ORAlteromonadaceae OR Azorhizobium OR Bdellovibrio OR Beggiatoa ORBordetella OR Brucella OR Burkholderiaceae OR Caulobacteraceae ORChryseobacterium OR Elizabethkingia OR Flavobacterium OR Francisella ORHalomonas OR Legionellaceae OR Leptothrix OR Methylobacillus ORMethylobacterium OR Methylococcaceae OR Methylophilus OR Methylosinus ORNeisseriaceae OR Ochrobactrum OR Paracoccus OR Pseudomonadaceae ORRhizobiaceae OR Taylorella OR Thermus OR Xanthobacter ORAcidaminococcaceae OR Bacteroides OR Bilophila OR Butyrivibrio ORDesulfovibrio OR Dichelobacter OR Fusobacterium OR Gram negative anaerobiccocci OR Oxalobacter OR Porphyromonas OR Prevotella OR ThaueraORThermotoga OR Wolinella OR Hyphomicrobium OR Nitrobacter ORNitrosomonadaceae OR Nitrosospira OR Aeromonadaceae OR ChromobacteriumOR Eikenella OR Enterobacteriaceae OR Pasteurellaceae OR Shewanella ORSphingomonas OR Streptobacillus OR Vibrionaceae OR Zymomonas ORLawsoniaOR Acidithiobacillus OR Afipia OR Alcaligenaceae OR Alteromonas ORAzorhizobium OR Bdellovibrio OR Bradyrhizobiaceae OR Bradyrhizobium ORBrucellaceae OR Burkholderiaceae OR Caulobacteraceae OR ComamonadaceaeOR Coxiellaceae OR Cytophagaceae OR Flavobacteriaceae OR Francisella ORGluconacetobacter OR Halomonadaceae OR Halothiobacillus OR klebsiella ORLegionellaceae OR Leptospiraceae OR Methylobacteriaceae ORMethylococcaceae OR Methylophilaceae OR Moraxellaceae OR Acinetobacter ORMoraxella OR Psychrobacter OR Neisseriaceae OR Nitrosomonadaceae OROchrobactrum OR Oxalobacteraceae OR Paracoccus OR Pedobacter ORPseudoalteromonas OR Pseudomonadaceae OR Rhizobiaceae ORRhodospirillaceae OR Rhodothermus OR Sphingobacterium OR SphingomonasORThermus OR Xanthobacter OR Xanthomonadaceae OR Zoogloea ORThiobacillusOR gramnegative OR "gram negative" OR gram-negative))) AND((((((antibiotic OR multidrug OR drug OR carbapenem) AND (resistant ORresistance)))) OR (((bacteria OR bacterial OR bacilli OR bacillus OR microbial ORbaumannii OR Acetobacteraceae OR Achromobacter OR Alcaligenes ORAlteromonadaceae OR Azorhizobium OR Bdellovibrio OR Beggiatoa ORBordetella OR Brucella OR Burkholderiaceae OR Caulobacteraceae ORChryseobacterium OR Elizabethkingia OR Flavobacterium OR Francisella OR Halomonas OR Legionellaceae OR Leptothrix OR Methylobacillus ORMethylobacterium OR Methylococcaceae ORMethylophilus OR Methylosinus ORNeisseriaceae OR Ochrobactrum OR Paracoccus OR Pseudomonadaceae ORRhizobiaceae OR Taylorella OR Thermus OR Xanthobacter ORAcidaminococcaceae OR Bacteroides OR Bilophila OR Butyrivibrio ORDesulfovibrio OR Dichelobacter OR Fusobacterium OR Gram negative anaerobiccocci OR Oxalobacter OR Porphyromonas OR Prevotella OR Thauera ORThermotoga OR Wolinella OR Hyphomicrobium OR Nitrobacter ORNitrosomonadaceae OR Nitrosospira OR Aeromonadaceae OR ChromobacteriumOR Eikenella OR Enterobacteriaceae OR Pasteurellaceae ORShewanella ORSphingomonas OR Streptobacillus OR Vibrionaceae OR Zymomonas ORLawsonia OR Acidithiobacillus OR Afipia OR Alcaligenaceae OR Alteromonas ORAzorhizobium OR Bdellovibrio OR Bradyrhizobiaceae OR Bradyrhizobium ORBrucellaceae OR Burkholderiaceae OR Caulobacteraceae OR ComamonadaceaeOR Coxiellaceae OR Cytophagaceae OR Flavobacteriaceae OR Francisella ORGluconacetobacter OR Halomonadaceae OR Halothiobacillus OR klebsiella ORLegionellaceae OR Leptospiraceae OR Methylobacteriaceae ORMethylococcaceae OR Methylophilaceae OR Moraxellaceae OR Acinetobacter ORMoraxella OR Psychrobacter OR Neisseriaceae OR Nitrosomonadaceae OROchrobactrum OR Oxalobacteraceae OR Paracoccus OR Pedobacter ORPseudoalteromonas OR Pseudomonadaceae OR Rhizobiaceae ORRhodospirillaceae OR Rhodothermus OR Sphingobacterium ORSphingomonasOR Thermus OR Xanthobacter OR Xanthomonadaceae OR ZoogloeaORThiobacillus OR gramnegative OR "gram negative" OR gram-negative) AND(resistant OR resistance)))) OR (((carbapenemase OR carbapenemases) AND(producer OR producers OR producing OR production)))))) AND ((therapy ORtreatment)))) AND ((((inprocess[sb])) OR (publisher[sb] NOTpubstatusnihms NOTpubstatuspmcsd NOT pmcbook))))) AND ((cure OR cured OR surviving ORsurvivor OR survivors OR survival OR healing OR healed OR outcome ORmortality OR death OR safety OR effectiveness OR successful OR success))))AND ((english[Language]) OR german[Language])) |
| **The Cochrane Library** | #1 | acetobacter* or achromobacter* or alcaligene* or alteromonadac* or azorhizobium or bdellovibrio orbeggiatoa or bordetella or brucella or burkholderiacea* or caulobacteracea* or chryseobacterium orelizabethkingia or flavobacterium or francisella or halomonas or klebsiella or legionellacea* or leptothrix ormethylobacill* or methylobacter* or methylococcacea* or methylophil* or methylosin* or neisseriacea* orochrobactrum or paracocc* or pseudomonadacea* or rhizobiacea* or taylorell* or thermus or xanthobacter oracidaminococcacea* or bacteroides or bilophila or butyrivibrio or desulfovibrio or dichelobacter orfusobacterium or oxalobacter or porphyromona* or prevotella or thauera or thermotoga or wolinella orhyphomicrobi* or nitrobacter or nitrosomonadacea* or nitrosospira or aeromonadacea* or chromobacteriumor eikenella or enterobacteriacea* or pasteurellacea* or shewanella or sphingomonas or streptobacill* orvibrionacea* or zymomonas or lawsonia or acidithiobacillus or afipia or alcaligenaceae or alteromonas orazorhizobium or bdellovibrio or bradyrhizobiaceae or bradyrhizobium or brucellaceae or burkholderiaceae orcaulobacteraceae or comamonadaceae or coxiellaceae or cytophagaceae or flavobacteriaceae or francisellaor gluconacetobacter or halomonadaceae or halothiobacillus or legionellaceae or leptospiraceae ormethylobacteriaceae or methylococcaceae or methylophilaceae or moraxellaceae or acinetobacter ormoraxella or psychrobacter or neisseriaceae or nitrosomonadaceae or ochrobactrum or oxalobacteraceae orparacoccus or pedobacter or pseudoalteromonas or pseudomonadaceae or rhizobiaceae orrhodospirillaceae or rhodothermus or sphingobacterium or sphingomonas or thermus or xanthobacter orxanthomonadaceae or zoogloea or thiobacillus or gramnegative or 'gram negative' or 'gram negative':ti,ab,kw(Word variations have been searched) |
|  | #2 | ((antibiotic or multidrug or drug or carbapenem) near/3 resistan*):ti,ab,kw or ((bacteria* or bacill* or microbialor baumannii) near/3 resistan*):ti,ab,kw or ((acetobacter* or achromobacter* or alcaligene* oralteromonadac* or azorhizobium or bdellovibrio or beggiatoa or bordetella or brucella or burkholderiacea* orcaulobacteracea* or chryseobacterium or elizabethkingia or flavobacterium or francisella or halomonas orklebsiella or legionellacea* or leptothrix or methylobacill* or methylobacter* or methylococcacea* ormethylophil* or methylosin* or neisseriacea* or ochrobactrum or paracocc* or pseudomonadacea* orrhizobiacea* or taylorell* or thermus or xanthobacter or acidaminococcacea* or bacteroides or bilophila orbutyrivibrio or desulfovibrio or dichelobacter or fusobacterium or oxalobacter or porphyromona* or prevotellaor thauera or thermotoga or wolinella or hyphomicrobi* or nitrobacter or nitrosomonadacea* or nitrosospira oraeromonadacea* or chromobacterium or eikenella or enterobacteriacea* or pasteurellacea* or shewanella orsphingomonas or streptobacill* or vibrionacea* or zymomonas or lawsonia or acidithiobacillus or afipia oralcaligenaceae or alteromonas or azorhizobium or bdellovibrio or bradyrhizobiaceae or bradyrhizobium orbrucellaceae or burkholderiaceae or caulobacteraceae or comamonadaceae or coxiellaceae orcytophagaceae or flavobacteriaceae or francisella or gluconacetobacter or halomonadaceae orhalothiobacillus or legionellaceae or leptospiraceae or methylobacteriaceae or methylococcaceae ormethylophilaceae or moraxellaceae or acinetobacter or moraxella or psychrobacter or neisseriaceae ornitrosomonadaceae or ochrobactrum or oxalobacteraceae or paracoccus or pedobacter orpseudoalteromonas or pseudomonadaceae or rhizobiaceae or rhodospirillaceae or rhodothermus orsphingobacterium or sphingomonas or thermus or xanthobacter or xanthomonadaceae or zoogloea orthiobacillus or gramnegative or 'gram negative' or 'gram negative') near/3 resistan*):ti,ab,kw (Word variationshave been searched) |
|  | #3 | (carbapenemase* near/3 produc* |
|  | #4 | #1 and (#2 or #3) |
|  | #5 | ((drug or medic*) near/3 (therapy or treatment)):ti,ab,kw or treatment or therapy:ti (Word variations have been searched) |
|  | #6 | #4 and #5 |

**Supplementary Table 1:** Advanced search strategy. Date of search: 2^nd^ December 2016

| **First author^[Ref]^**  **Year** | **Study**  **design** | **Microorganism** | **Disease** | **Monotherapy** | **Combination therapy** |
| --- | --- | --- | --- | --- | --- |
| Balkan[^17^](#_ENREF_17)  2014 | CCS | OXA-48-like carbapenemase-producing *Entero-bacteriaceae* | BSI | Carbapenem | Combinations of colistin, aminoglycosides, carbapenems, cephalosporins and tigecycline |
| Batirel[^18^](#_ENREF_18)  2014 | CHS | XDR-*Acinetobacter baumannii* | BSI | Colistin with or without sulbactam | Colistin plus (carbapenem or tigecycline or amikacin or netilmicin or genatmicin or rifampicin or piperacillin/tazobactam) |
| Bergamasco[^33^](#_ENREF_33)  2012 | CS | Carbapenmase (KPC)-producing *Klebsiella pneumoniae* | Mixed (incl. pneumonia, BSI, UTI, SSI) | Imipenem or polymyxin B | Various combinations of imipenem, meropenem, polymyxin B and tigecycline |
| Cai[^34^](#_ENREF_34)  2016 | CHS | XDR Gram-negative bacteria | Mixed (incl. CNS-, GI-infection, pneumonia, tracheo-bronchitis, SSTI, BJI, UTI, BSI, bacteriemia) | Polymyxin | Polymyxin plus (carbapenem or fluoroquinolone or beta-lactam/beta-lactamase inhibitor or aminoglycoside or cephalosporin or aztreonam or rifampicin or tigecycline) |
| Cetin[^35^](#_ENREF_35)  2016 | CHS | MDR and XDR Gram-negative bacteria | Mixed (incl. RTI, BSI, UTI, SSI, others) | Colistin | Colistin plus (carbapenem or Betalactam or aminoglycosides or quinolones or tigecycline) |
| Daikos[^19^](#_ENREF_19)  2009 | CHS | VIM-1 Metallo-β-lactamase-producing  *Klebsiella pneumoniae* | BSI | Meropenem or imipenem or colistin or aminoglycoside | Colistin or Aminoglycoside with Carbapenem (meropenem or imipenem) |
| Daikos[^20^](#_ENREF_20)  2014 | CHS | Carbapenemase (KPC, VIM)-producing *Klebsiella pneumoniae* | BSI | Tigecycline or colistin or aminoglycoside or carbapenem or other | Various combinations of the drugs used as monotherapy |
| Durante-Mangoni[^36^](#_ENREF_36)  2013 | RCT | XDR- *Acinetobacter baumannii* | Mixed (incl. pneumonia, BSI, IAI) | Colistin | Colistin plus rifampicin |
| Falagas[^37^](#_ENREF_37)  2006 | CS | Polymyxin-only-susceptible Gram-negative bacteria | Mixed (incl. pneumonia, BSI, UTI, SSTI, BJI, IAI, CNS-infection) | Colistin | Colistin plus (piperacillin/tazobactam or meropenem or ampicillin or ceftazidime or ambicillin/sulbactam or ceftriaxone) |
| Falagas[^38^](#_ENREF_38)  2010 | CHS | MDR Gram-negative bacteria | Mixed (incl. pneumonia, BSI, UTI, SSTI, BJI, IAI, CNS- infection) | Colistin | Colistin plus (meropenem or piperacillin/tazobactam or ampicillin/sulbactam or other) |
| Freire[^21^](#_ENREF_21)  2015 | CHS | XDR-*Acinetobacter baumannii* | BSI (CLABSI, pneumonia, IAI, BJI, SSTI) | Polymyxin B or Polymyxin E | (Polymyxin plus aminoglycoside) or (polymyxin plus ampicillin/sulbactam) |
| Furtado[^7^](#_ENREF_7)  2007 | CCS | MDR-*Pseudomonas aeruginosa* | HAP | Polymyxcin B | Polymyxcin B plus (imipenem or ciprofloxacin or cefepime or ceftazidime) |
| Goff[^39^](#_ENREF_39)  2014 | CHS | MDR- *Acinetobacter baumannii* | Mixed (incl. pneumonia, BSI, UTI, SSI, IAI, BJI) | Minocycline | Minocycline plus (colistin or (colistin and doripenem) or ampicillin/sulbactam or (colistin and doripenem and ampicillin/sulbactam) or (doripenem and ampicillin/sulbactam) or (colistin and ampicillin/sulbactam) |
| Gonzales-Padilla[^40^](#_ENREF_40)  2015 | CHS | Carbapenem-resistant and colistin-resistant *Klebsiella pneumoniae* | Sepsis due to various causes (incl. pneumonia, BSI, UTI, SSTI, IAI, CNS-infection, others) | Tigecycline or gentamicin | Tigecycline plus gentamicin |
| Hachem[^41^](#_ENREF_41)  2007 | CHS | MDR- *Pseudomonas aeruginosa* | Mixed (incl. pneumonia, BSI, UTI, SSTI) | Colistin or single antipseudomonal agent | Colistin plus (aminoglycoside or carbapenem or cephalosporin or fluoroquinolone or antipseudomonal penicillin) |
| He[^8^](#_ENREF_8)  2016 | CHS | XDR-*Acinetobacter baumannii* | VAP | Carbapenem or cefoperazone/sul-bactam | Tigecycline plus carbapenem and/or cefoperazone/sulbactam |
| Hernández-Torres[^42^](#_ENREF_42)  2012 | CHS | MDR- and carbapenem-resistant *Acinetobacter baumannii* | Mixed (incl. pneumonia, BSI, SSTI, UTI, IAI, CNS-infections) | Tigecycline or amikacin or amikacin/sul-bactam or tobramycin or colistin | Combinations of tigecycline, rifampicin, amikacin, colistin |
| Jean[^9^](#_ENREF_9)  2016 | CCS | XDR- *Acinetobacter baumannii* | VAP with bacteremia | Imipenem/sul-bactam | Tigecycline plus imipenem |
| Ji[^43^](#_ENREF_43)  2015 | RCT | KPC-producing *Klebsiella pneumoniae* | Mixed (source of bacteria: sputum, peritoneal fluid, blood, urine, CSF) | Tigecycline | (Cefepime plus amoxicillin/clavulanic acid plus fosfomycin) or (tigecyclin plus fosfomycin or amikacin or cefepime or cefoperazone/sulbactam or meropenem) |
| Katsiari[^44^](#_ENREF_44)  2015 | CHS | Carbapenemase (KPC, VIM)-producing *Klebsiella pneumoniae* | Mixed (incl. bacteremia, VAP, SSI, IAI, SSTI) | Tigecyclin or colistin | Various combinations of tigecycline, colistin, aminoglycoside, cefepime, carbapenem, fosfomycin |
| Kuo[^22^](#_ENREF_22)  2007 | CHS | MDR- *Acinetobacter*  *baumannii* | BSI | Carbapenem | Carbapenem plus (amikacin or ampicillin/sulbactam) |
| Lee[^45^](#_ENREF_45)  2005 | CHS | PDR-  *Acinetobacter baumanii* | Mixed | Carbapenem/sul-bactam | 2nd or 3rd generation cephalosporin, antipseudomonas penicillin, or fluoroquinolone, all in combination with amikacin |
| Lim[^23^](#_ENREF_23)  2011 | CHS | MDR- *Acinetobacter* species | BSI | Colistin | Colistin plus (carbapenem or other) |
| Linden[^46^](#_ENREF_46)  2003 | CHS | MDR-  *Pseudomonas aeruginosa* | Mixed (incl. pneumonia,  endocarditis, BSI, SSTI, IAI, others) | Colistin | Colistin plus (amikacin or antipseudomonal beta-lactam) |
| Lopez-Cortes[^47^](#_ENREF_47)  2014 | CHS | MDR-  *Acinetobacter baumannii* | Sepsis due to UTI, SSTI, pneumonia, IAI, others) | Colistin or carbapenem or tigecycline or tetracycline or colistin/sulbactam | Various combinations of colistin, carbapenem, tigecycline, rifampicin, aminoglycoside |
| Navarro-San Francisco[^24^](#_ENREF_24)  2012 | CHS | OXA-48-carbapenemase-producing  *Entero-bacteriaceae* | BSI | Amikacin or tigecycline or colistin | Colistin plus (tigecycline or meropenem or amikacin or fosfomycin) or (tigecycline plus (fosfomycin or amikacin)) or (meropenem plus amikacin) or (ceftriaxone plus ciprofloxacine) or (amikacin plus ciprofloxacin) |
| Papadimitriou-Olivgeris[^25^](#_ENREF_25)  2014 | CHS | Carbapenemase (KPC)-producing *Klebsiella pneumoniae* | BSI | one active antibiotic | At least two active antibiotics |
| Parchem[^10^](#_ENREF_10)  2016 | CHS | MDR-  Gram-negative Bacteria | Pneumonia | Colistin | Colistin plus (tigecycline or minocycline or ampicillin/sulbactam or imipenem/cilastatin or doripenem or other) |
| Qureshi[^26^](#_ENREF_26)  2012 | CHS | Carbapenemase (KPC)-producing *Klebsiella pneumoniae* | Bacteremia due to various causes (incl. pneumonia, BSI, UTI) | Colistin/polymyxin B or tigecycline or carbapenem or gentamicin or ampicillin/sul-bactam or piperacillin/tazo-bactam | (Colistin/polymyxin B plus (tigecycline or carbapenem or fluoroquinolone)) or (tigecycline plus (carbapenem or aminoglycoside)) or (carbapenem plus fluoroquinolone) or (aztreonam plus fluoroquinolone) or (cefepime plus gentamicin) |
| Ribera[^32^](#_ENREF_32)  2015 | CHS | MDR- *Pseudomonas aeruginosa* | BJI | Colistin or beta-lactam | (Colistin plus beta-lactam) or (amikacin plus beta-lactam) |
| Rigatto[^48^](#_ENREF_48)  2015 | CHS | XDR- *Acinetobacter baumannii* or *Pseudomonas aeruginosa* | Mixed (incl. pneumonia, BJI, UTI, IAI) | Polymyxin B | (Polymyxin B plus (carbapenem or ampicillin/sulbactam or piperacillin/tazobactam or rifampicin or amikacin) |
| Rihani[^49^](#_ENREF_49)  2012 | CHS | Carbapenemase-producing  *Entero-bacteriaceae* | Mixed (incl. pneumonia, BSI, SSTI, UTI) | Colistin or meropenem or aminoglycosides or tigecycline | (Colistin plus (meropenem or tigecycline or rifampin or amikacin/meropenem/tige-cycline)) or (meropenem plus amikacin) or (tigecyclin plus amikacin plus cefepime) or (tobramycin plus cefepime) |
| Sánchez-Romero[^50^](#_ENREF_50)  2012 | CHS | VIM-1-producing *Klebsiella pneumoniae* | Mixed (incl. pneumonia, BSI, SSTI, UTI, IAI, meningitis) | Tigecycline or meropenem or ertapenem or amikacin | (Tigecycline plus colistin) or (tigecycline plus colistin plus amikacin) |
| Schafer[^51^](#_ENREF_51)  2007 | CS | MDR- *Acinetobacter baumannii* | Mixed (VAP and/or bacteremia) | Tigecycline | (Tigeycline plus imipenem) or (tigecyline plus imipenem plus colistin) |
| Shields[^52^](#_ENREF_52)  2012 | CHS | XDR- *Acinetobacter baumannii* | Mixed (RTI or bacteremia) | Cefepime or tigecycline or carbapenem | Colistin plus (tigecycline or tigecycline/rifampcin or tigecycline/amikacin or tigecycline/ampicillin/sul-bactam or rifampicin or cefepime or ampicillin/sulbactam or carbapenem/ampicillin/sul-bactam or carbapenem/tigecycline or carbapenem/rifampicin) or (carbapanem plus ampicillin/sulbactam) |
| Simsek[^53^](#_ENREF_53)  2012 | CCS | Colistin-only susceptible *Acinetobacter baumannii* | Mixed (including pneumonia, BSI, UTI, SSI, IAI, meningitis) | Colistin | Colistin plus (rifampicin or carbapenem or tigecyclin or ampicillin/sulbactam or cefoperazone/sulbactam or (tigecycline plus rifampicin) or (carbapanem plus rifampicin)) |
| Souli[^54^](#_ENREF_54)  2008 | CS | VIM-1 metallo–  β-lactamase  producing  *Entero-bacteriaceae* | RTI or bacteremia | Colistin or tigecycline | Various combinations of colistin, imipenem, gentamicin, doxycycline, piperacillin/tazobactam, amikacin, meropenem |
| Tasbakan[^11^](#_ENREF_11)  2011 | CHS | MDR- *Acinetobacter baumannii* | Pneumonia | Tigecycline | Tigecyline plus (cefoperazone/sulbactam or netilimicin or amikacin) |
| Tofas[^27^](#_ENREF_27)  2016 | CHS | Carbapenemase (VIM or KPC)-producing *Klebsiella pneumoniae* | BSI | Colistin or gentamicin | (Gentamicin plus tigecycline) or (gentamicin plus colistin) or (colistin plus gentamicin plus tigecycline) or (colistin plus gentamicin plus doripenem) or (colistin plus gentamicin plus fosfomycin) |
| Torres-Gonzalez[^55^](#_ENREF_55)  2016 | CCS | OXA-232 carbapenem-resistant *Enterobacteriaceae* | Mixed (including UTI, IAI, pneumonia, SSTI) | Fosfomycin or meropenem or nitrofurantoin or ciprofloxacin or ceftriaxone or imipenem | (Meropenem plus ciprofloxacin) or (imipenenem plus gentamicin) or (meropenem plus amikacin) or (meropenem plus SMX/TMP) or (piperacilline/tazobactam plus SMX/TMP) or (ertapenem plus ciprofloxacin) |
| Tseng[^28^](#_ENREF_28)  2007 | CHS | XDR-  *Acinetobacter baumannii* | BSI | Ampicillin/sul-bactam | Carbapenem and aminoglycosides and/or ampicillin/sulbactam |
| Tsioutis[^12^](#_ENREF_12)  2016 | CHS | XDR *Acinetobacter baumannii* | VAP | Colistin | (Colistin plus tigecycline) or (colistin plus carbapenem) or (colistin plus tigecycline plus carbapenem) |
| Tumbarello[^29^](#_ENREF_29)  2012 | CHS | Carbapenemase (KPC)-producing *Klebsiella pneumoniae* | BSI | Colistin or tigecycline or gentamicin | Various combinations of colistin, tigecycline, gentamicin, meropenem |
| Tumbarello[^56^](#_ENREF_56)  2015 | CHS | Carbapenemase (KPC)-producing *Klebsiella pneumoniae* | Mixed (including pneumonia, BSI, UTI, IAI, others) | Colistin or tigecycline or gentamicin | Various combinations of agents used as monotherapy and carbapenems and rifampicin |
| Vergara-Lopez[^57^](#_ENREF_57)  2015 | CHS | Metallo-β-lactamase-producing  *Klebsiella oxytoca* | Mixed (including pneumonia, BSI, UTI, IAI) | Amikacin or tigecycline | Amikacin plus (tigecycline or imipenem or fosfomycin) |
| Wang[^30^](#_ENREF_30)  2017 | CHS | MDR  Gram-negative bacteria | BSI | Cephalosporins or carbapenems or beta-lactam/beta-lactamase inhibitor or fluororquinolones | (Cephalosporin plus (aminoglycoside or fluoroquinolone)) or (carbapenem plus (fluoroquinolone or beta-lactam/beta-lactamase inhibitor)) or (cephalosporin plus carbapenem plus others) |
| Wood[^13^](#_ENREF_13)  2003 | CS | MDR-  *Acinetobacter baumannii* | VAP | Minocycline or doxycycline | (Minocycline or doxycycline) plus (imipenem or amikacin or trovafloxaxin/TMP/SMX or ampicilin/sulbactam or amikacin/trovafloxacin) |
| Wu[^58^](#_ENREF_58)  2014 | CHS | MDR- *Acinetobacter baumannii* | Mixed | Tigecycline or tigecycline/sul-bactam | Tigecycline plus (colistin or cefoperazone/sulbactam or carbapenem or fosfomycin) or (tigecycline plus colistin plus fosfomycin) or (tigecycline plus colistin plus carbapenem) or (tigecycline plus colistin plus cefoperazon/sulbactam) |
| Ye[^14^](#_ENREF_14)  2011 | CHS | MDR-  *Acinetobacter baumannii* | Pneumonia | Tigecycline with or without sulbactam | Tigecycline plus (cephalosporin or colistin or carbapenem or amikacin or fluoroquinolone) |
| Ye[^15^](#_ENREF_15)  2016 | CCS | MDR *Acinetobacter calcoaceticus-Acinetobacter baumannii* complex | Pneumonia | Tigecyclin | Tigecyclin plus (cephalosporin or colistin or carbapenem or aminoglycoside or fluoroquinolone) |
| Yilmaz[^16^](#_ENREF_16)  2015 | CHS | MDR and XDR *Acinetobacter baumannii* | VAP | Colistin with or without sulbactam | Colistin plus carbapenem |
| Zarkotou[^31^](#_ENREF_31)  2011 | CHS | Carbapenemase (KPC)-producing *Klebsiella pneumoniae* | BSI | Colistin or tigecyclin or gentamicin or carbapenem | (Tigecycline plus (colistin or gentamicin or carbapenem or (colistin plus carbapenem) or (colistin plus gentamicin) or amikacin)) or (colistin plus gentamicin) or (carbapenem plus gentamicin) |
| Zavascki[^59^](#_ENREF_59)  2006 | CHS | Metallo-β-lactamase-producing  *Pseudomonas aeruginosa* | Mixed (including pneumonia, BSI, SSTI, UTI, IAI, other) | Polymyxin B or aztreonam or piperacillin/tazo-bactam | Combinations of agents used for monotherapy, amikacin and beta-lactams |

**Supplementary Table 2**: Study characteristics

Abbreviations: CCS, case-control study; CHS, cohort study; CS, case series; RCT, Randomized controlled trial; MDR, multidrug-resistant; XDR, extensively drug-resistant; PDR, pan-drug resistant; BSI, Bloodstream infections; UTI, urinary tract infections; SSI, surgical site infections; CNS, central nervous system; SSTI, skin and soft tissue infection; BJI, bone and joint infection; GI, gastrointestinal; RTI, respiratory tract infection; IAI, intraabcominal infection; HAP, hospital acquired pneumonia; CLABSI, central line associated blood stream infection; VAP, Ventilator-associated pneumonia; CSF, cerebro-spinal fluid


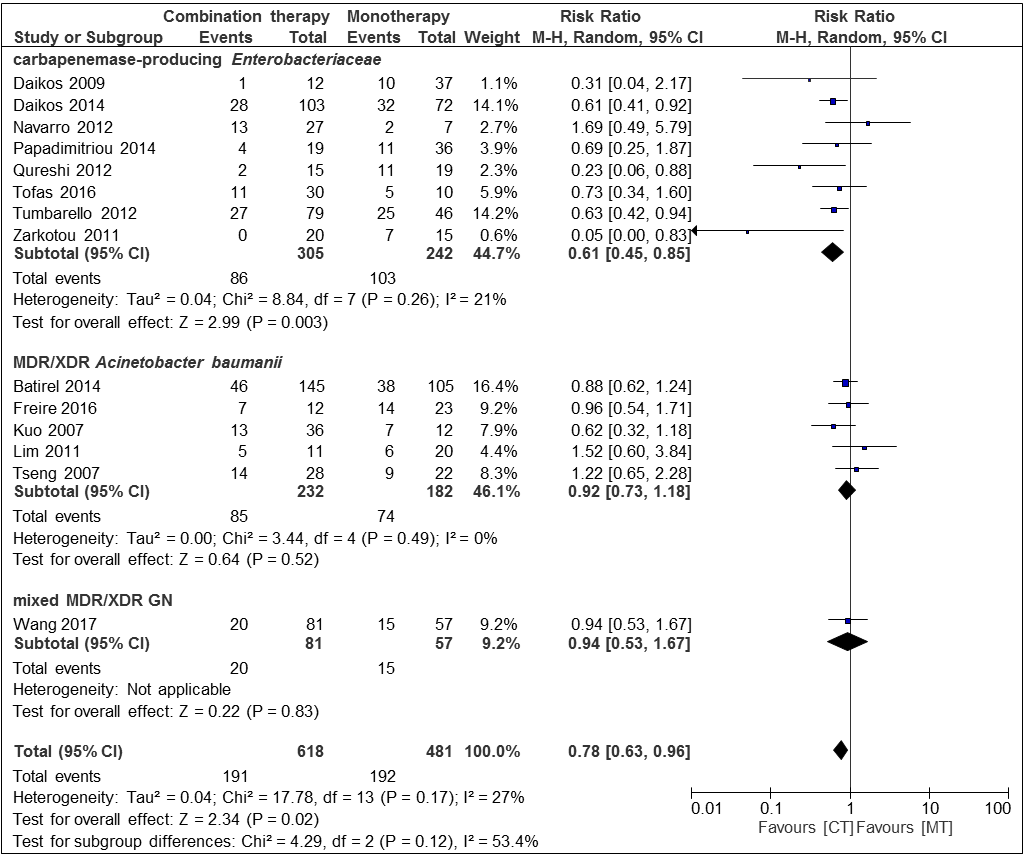


**Supplementary Figure 1:** Risk ratios for mortality rates of case series and cohort studies of blood stream infections stratified by different bacteria. Data markers indicate Risk ratios and error bars indicate 95% CIs


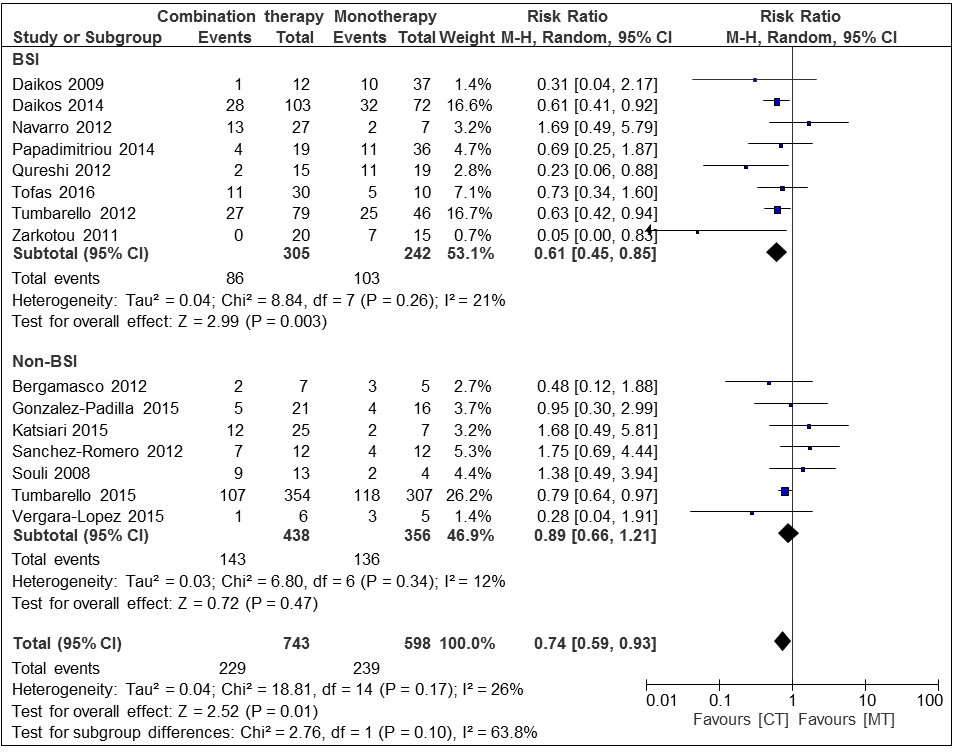


**Supplementary Figure 2:** Risk ratios for mortality rates of case series and cohort studies including infections caused by carbapenemase-producing Enterobacteriaceae only, stratified by bloodstream infections (BSI) and non-bloodstream infections (Non-BSI, i.e. mixed infections). Data markers indicate Risk ratios and error bars indicate 95% Cis


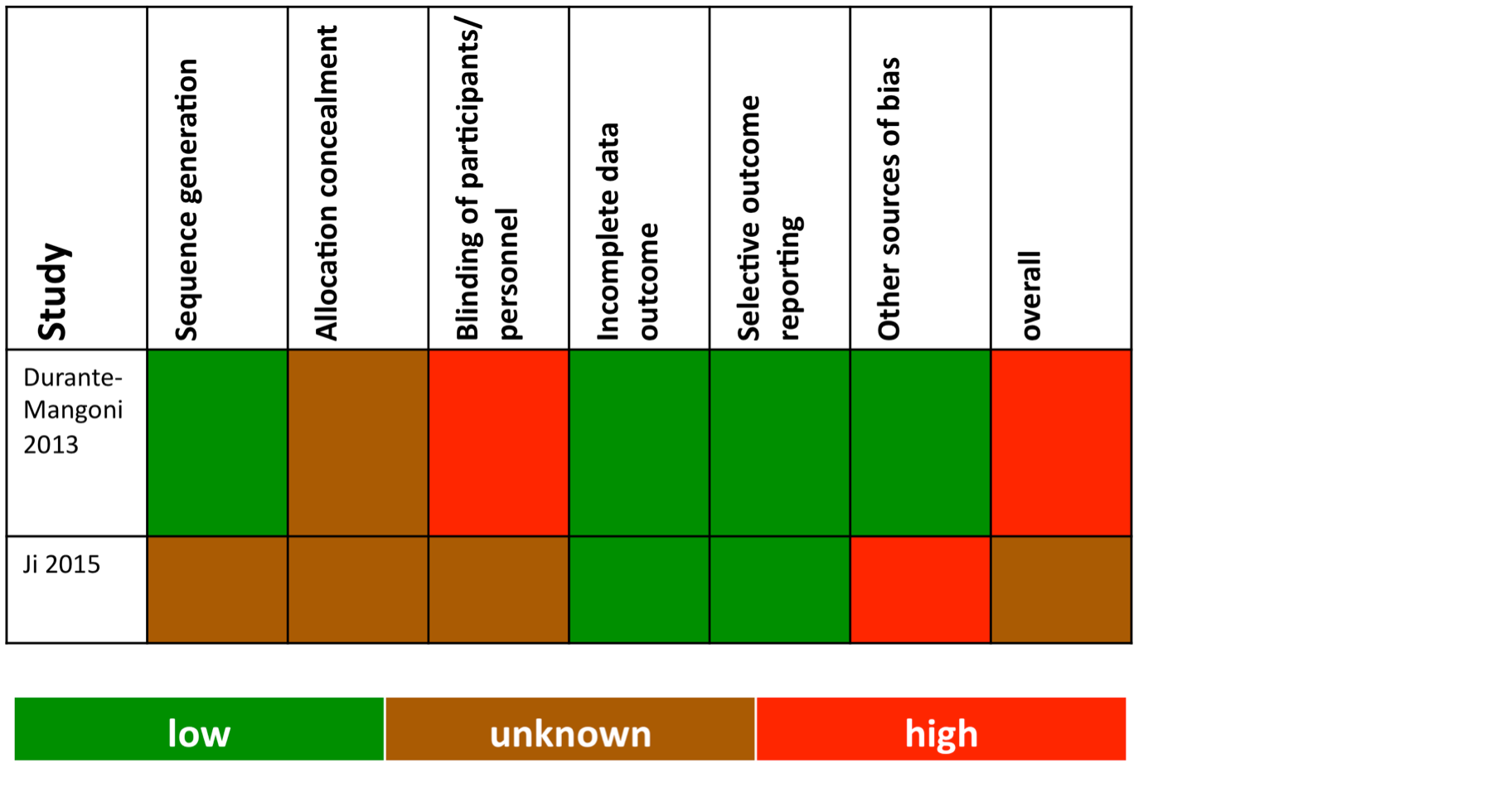


**Supplementary Table 3:** Risk of bias in randomized controlled trials

| Study | Selection | Comparability | Exposure | Quality |
| --- | --- | --- | --- | --- |
| Balkan 2014 | *** |  | ** | Poor |
| Furtado 2007 | *** |  | ** | Poor |
| Jean 2016 | *** | ** | *** | Good |
| Simsek 2012 | *** |  | ** | Poor |
| Torres-Gonzalez 2016 | *** |  | ** | Poor |
| Ye 2016 | *** |  | ** | Poor |

**Supplementary Table 4:** Risk of bias in case-control studies

| **Study** | **Selection** | **Comparability** | **Outcome** | **Quality** |
| --- | --- | --- | --- | --- |
| Batirel 2014 | ***** | ** | *** | Good |
| Bergamasco 2012 | ***** |  | *** | Poor |
| Cai 2016 | ***** | ** | *** | Good |
| Cetin 2016 | ***** |  | *** | Poor |
| Daikos 2009 | ***** |  | *** | Poor |
| Daikos 2014 | ***** | ** | *** | Good |
| Falagas 2006 | ***** |  | *** | Poor |
| Falagas 2010 | ***** | ** | *** | Good |
| Freire 2015 | ***** |  | *** | Poor |
| Goff 2014 | ***** |  | *** | Poor |
| Gonzales-Padilla 2015 | ***** |  | *** | Poor |
| Hachem 2007 | ***** |  | *** | Poor |
| He 2016 | ***** |  | *** | Poor |
| Hernández-Torres 2012 | ***** | ** | *** | Good |
| Katsiari 2015 | ***** |  | *** | Poor |
| Kuo 2007 | ***** |  | *** | Poor |
| Lee 2005 | ***** |  | *** | Poor |
| Lim 2011 | ***** |  | *** | Poor |
| Linden 2003 | ***** |  | *** | Poor |
| Lopez-Cortes 2014 | ***** | ** | *** | Good |
| Navarro-San Francisco 2012 | ***** |  | *** | Poor |
| Papadimitriou-Olivgeris 2014 | ***** | ** | *** | Good |
| Parchem 2016 | ***** | ** | *** | Good |
| Qureshi 2012 | ***** | ** | *** | Good |
| Ribera 2015 | ***** |  | *** | Poor |
| Rigatto 2015 | ***** | ** | *** | Good |
| Rihani 2012 | ***** |  | *** | Poor |
| Sánchez-Romero 2012 | ***** |  | *** | Poor |
| Schafer 2007 | ***** |  | *** | Poor |
| Shields 2012 | ***** |  | *** | Poor |
| Souli 2008 | ***** |  | *** | Poor |
| Tasbakan 2011 | ***** |  | *** | Poor |
| Tofas 2016 | ***** | ** | *** | Good |
| Tseng 2007 | ***** | ** | *** | Good |
| Tsioutis 2016 | ***** | ** | *** | Good |
| Tumbarello 2012 | ***** | ** | *** | Good |
| Tumbarello 2015 | ***** | ** | *** | Good |
| Vergara-Lopez 2015 | ***** |  | *** | Poor |
| Wang 2017 | ***** | ** | *** | Good |
| Wood 2003 | ***** |  | *** | Poor |
| Wu 2014 | ***** |  | *** | Poor |
| Ye 2011 | ***** | ** | *** | Good |
| Yilmaz 2015 | ***** |  | *** | Poor |
| Zarkotou 2011 | ***** |  | *** | Poor |
| Zavascki 2006 | ***** |  | *** | Poor |

**Supplementary Table 5:** Risk of bias in case series and cohort studies
